# The liquid-glass-jamming transition in disordered ionic nanoemulsions

**DOI:** 10.1038/s41598-017-13584-w

**Published:** 2017-11-08

**Authors:** Marco Braibanti, Ha Seong Kim, Nesrin Şenbil, Matthew J. Pagenkopp, Thomas G. Mason, Frank Scheffold

**Affiliations:** 1 Department of Physics, University of Fribourg, 1700 Fribourg, Switzerland; 2 Department of Chemistry and Biochemistry, University of California, Los Angeles, California, 90095 USA; 3 Department of Physics, University of Fribourg, 1700 Fribourg, Switzerland; 4 Department of Chemistry and Biochemistry, University of California, Los Angeles, California, 90095 USA; 5 Department of Chemistry and Biochemistry, and Department of Physics and Astronomy, University of California, Los Angeles, California, 90095 USA; 6 Department of Physics, University of Fribourg, 1700 Fribourg, Switzerland

## Abstract

In quenched disordered out-of-equilibrium many-body colloidal systems, there are important distinctions between the glass transition, which is related to the onset of nonergodicity and loss of low-frequency relaxations caused by crowding, and the jamming transition, which is related to the dramatic increase in elasticity of the system caused by the deformation of constituent objects. For softer repulsive interaction potentials, these two transitions become increasingly smeared together, so measuring a clear distinction between where the glass ends and where jamming begins becomes very difficult or even impossible. Here, we investigate droplet dynamics in concentrated silicone oil-in-water nanoemulsions using light scattering. For zero or low NaCl electrolyte concentrations, interfacial repulsions are soft and longer in range, this transition sets in at lower concentrations, and the glass and the jamming regimes are smeared. However, at higher electrolyte concentrations the interactions are stiffer, and the characteristics of the glass-jamming transition resemble more closely the situation of disordered elastic spheres having sharp interfaces, so the glass and jamming regimes can be distinguished more clearly.

## Introduction

The glass and jamming transitions of colloidal repulsive hard and soft spheres have been the subject of numerous studies over the last decades. These studies have been conducted in order to address fundamental questions related to the microscopic mechanisms of crystallization and glass formation in condensed matter physics^1–8^. The crystalline phase has the lowest free energy and is in thermodynamic equilibrium, but a long-lived amorphous glassy state is readily obtained whenever crystallization is frustrated by a nonuniform particle size distribution or by a rapid quench to raise the volume fraction of the dispersed particles, ${\varphi }$. In the case of a rapid quench of monodisperse hard spheres, the standard scenario suggests that for a volume fraction of spheres associated with a glass transition, ${{\varphi }}_{{\rm{g}}}\simeq 0.58$, a weak glassy state is formed, the viscosity diverges $\eta \to \infty$, and a finite low-frequency elastic shear modulus develops, ${G}_{0}\sim {k}_{B}T/{R}^{3}$, for spheres having an average radius $R$. Further increasing the density, the particles can touch and jam, forming a space filling network of contact forces^9^. Contact forces are typical of granular materials composed of non-colloidal grains, but are not strictly valid for stable dispersions of colloidal particles in a viscous liquid. In such colloidal dispersions, the particles diffuse under the influence of thermal fluctuations and are stabilized against irreversible aggregation by short-range slippery repulsive interactions that preclude strict solid-solid frictional contact in quiescent conditions. Often, thermal or Brownian fluctuations are justifiably neglected in non-colloidal granular systems, yet these fluctuations are a measure for the influence of entropy and can be important in colloidal soft matter composed of dispersed spherical objects, including dispersions of hard nanospheres as well as nanoemulsions of deformable droplets of one liquid (e.g. oil) dispersed in an immiscible liquid (e.g. water) stabilized against coalescence by adsorbed surfactant molecules.

The glass transition can be understood by the formation and fluctuation of long-lived cages of particles over many length scales; a disordered system of monodisperse spheres can be formed by raising ${\varphi }$ rapidly enough above a value ${{\varphi }}_{{\rm{g}}}$ to preclude the colloidal disorder-order transition known for hard spheres^10,11^, and the disordered glassy system self-adjusts given the available space and maximizes entropy subject to non-equilibrium constraints which can preclude crystal formation. By contrast, jamming is associated with direct repulsive particle-particle interactions, leading to an increase in the internal energy as ${\varphi }$ is raised beyond a jamming point ${{\varphi }}_{{\rm{J}}}$ at which interparticle repulsions arising from this crowding begin to play a significant role. In early work, this jamming point of constituent objects has also been referred to as a critical volume fraction, ${{\varphi }}_{{\rm{c}}}$, in the context of monodisperse emulsions^12,13^, which clearly showed an onset of elasticity at ${{\varphi }}_{{\rm{c}}}$ associated with the volume fraction of jamming, ${{\varphi }}_{{\rm{J}}}\simeq 0.64$ ^13–15^, a similar value as that previously suggested for the random close packing of monodisperse spheres^16^. For hard spheres, upon approaching the jamming point ${{\varphi }}_{{\rm{J}}}$, the free volume available for translation per sphere vanishes^17^ and the shear elastic modulus diverges ${G}_{0}\to \infty$ at ${{\varphi }}_{{\rm{J}}}\simeq 0.64$ ^14,15^. For deformable spheres this divergence is avoided, and the jamming transition leads to a strong but less abrupt rise in the shear modulus. The phenomenon of jamming has been identified experimentally in the prototypical soft matter system of uniform deformable droplets: in experiments on the shear rheology of monodisperse colloidal emulsions^12^, analytical modeling of these experiments involving near-equilibrium energy minimization^12,18,19^, and computer simulations^13,20^. More recent recent studies on emulsions and soft microgels as well as granular media have made attempts to verify predictions by computer simulations^18,21–27^.

Over the last decade, the distinction between the entropic glass and the jamming transitions has been discussed controversially^28–30^, despite a bulk of previous experimental studies on disordered monodisperse spherical colloids dating back to the late 1980’s^1,2,31–33^ as well as more recent work on nanoemulsions^34^ and numerical studies^7^. A high-resolution experimental study covering the dynamics and degree of confinement of particle or droplets covering the range of ${\varphi }$ below, through, and above these two transitions, which could potentially settle these questions experimentally, has not been performed thus far.

Recently, a near-equilibrium energy minimization approach has been introduced to model the entropic, electrostatic, and interfacial contributions to the free energy of a dense disordered system of droplets from the glassy regime just below jamming through and above the jamming point^35^. By taking appropriate derivatives of this free energy, this entropic-electrostatic-interfacial (EEI) model can then be used to predict the osmotic pressure and low-frequency shear elastic moduli of ionic colloidal emulsions and nanoemulsions^35^. This model accurately predicts the measured ${\varphi }$-dependent plateau elastic shear moduli, ${G}_{{\rm{p}}}$ of colloidal droplets stabilized by Debye screened-charge repulsions over more than four orders of magnitude for droplet radii ranging from about 25 nm to about 1 micron^35^. Moreover, computer simulations and calculations^7^ have treated jamming of soft colloidal objects and also show a less-abrupt transition in $G({\varphi })$ near ${{\varphi }}_{{\rm{J}}}$. For soft spheres, the dynamic and rheological properties in the glassy regime ${\varphi }\ge {{\varphi }}_{{\rm{g}}}\to {{\varphi }}_{{\rm{J}}}\to 1$ are then controlled by a complex interplay between entropic contributions and energetic contributions to the free energy. Thus far, this correlated two-step transition for soft objects (i.e. glass transition and then jamming transition) as ${\varphi }$ is raised has a eluded a full theoretical treatment. In addition, experiments that clearly reveal the low-frequency dynamics and relaxations, the ergodic-nonergodic transition, and the behavior of the plateau shear modulus with adequate resolution in ${\varphi }$ below, near, and above ${{\varphi }}_{{\rm{g}}}$ and ${{\varphi }}_{{\rm{J}}}$ have been lacking until now. This is especially true for submicron sized solid colloids mimicking the behaviour of hard spheres where it has been argued recently that systematic errors of ${\rm{\Delta }}{\varphi }\ge 0.03$ in determining the volume fraction ${\varphi }$ ‘are probably unavoidable’^36^. In this regard, incompressible yet deformable emulsion droplets hold a key advantage over many other kinds of soft matter systems that can jam, because the droplet volume fraction can be measured directly and accurately, rather than being inferred indirectly, as is typically the case for other compressible soft objects. Moreover, the jamming transition can be crossed reversibly without any hysteresis and therefore concentration series covering a range of volume fractions from $0 < {\varphi } < 0.9$ can be prepared with an accuracy of better than ${\rm{\Delta }}{\varphi }\le 0.005$ ^8,12,34^.

In the present work, we use dynamic light scattering to study the transition from a supercooled liquid to a glass and subsequently to a jammed state as ${\varphi }$ is raised in charge-stabilized size-fractionated nanoemulsions that have tunable repulsive interactions. For relatively stiff repulsive interaction potentials, there is a possibility of distinguishing between the glass transition and the jamming transition; whereas, for softer repulsive interaction potentials, these two transitions become increasingly smeared together, so measuring a clear distinction between where the glass ends and where jamming begins, based on observables such as the time-dependent particle mean square displacement^37^ (or in analogy, the frequency-dependent shear modulus or the yield stress^7,19^), becomes very difficult or even impossible. In order to dial between these two different interaction regimes, we control the stiffness of the repulsive interaction potential between droplets in size-fractionated nanoemulsions by screening repulsive forces using an added non-amphiphilic electrolyte, NaCl, thereby reducing the range of droplet-droplet interactions through the Debye length ${\lambda }_{{\rm{D}}}$.

## Results and Discussion

### Nanoemulsions

We initially produce an unfractionated nanoemulsion having a significant polydispersity using a high pressure microfluidic homogenizer device (Microfluidics Inc. M-110P, 75 micron Y-type interaction chamber). This polydisperse nanoemulsion is then repeatedly fractionated using an ultracentrifugal separation method to provide a uniform droplet size: radius $\langle R\rangle =130$ nm, further denoted as R for brevity, and polydispersity 12% (standard deviation $\delta R$ divided by the mean $< R >$), Figures S1 and S2 (a) ^34^. While fractionating, an anionic amphiphilic surfactant, sodium dodecyl sulfate (SDS, MP Biomedicals, Ultrapure) in excess of its critical micelle concentration (CMC) $\,\simeq \,8$ mM is maintained to ensure droplet stability. Through this repeated centrifugal fractionation process, the SDS concentration is also fixed at 10 mM, just above the CMC, to ensure strong stability against droplet coalescence over the range of ${\varphi }$ we explore but also to preclude strong micellar depletion attractions between droplets. The product of this fractionation process is a jammed disordered elastic nanoemulsion of uniform droplets having an elastic shear modulus in excess of 1 kPa.

This master nanoemulsion sample at high ${\varphi }$ is used to prepare a set of disordered nanoemulsions at different lower ${\varphi }$ by means of dilution with a 10 mM SDS solution (SDS, Sigma Aldrich, purity $\ge$98.5% (GC)) with an accuracy better than $\sim 0.005$ in volume fraction ${\varphi }$ through the use of a balance. Stock solutions having different amounts of sodium chloride salt NaCl ($\ge$99%, ReactoLab, pharmaceutical grade) are prepared by diluting the solution with the desired ionic strength and then ultracentrifuging again. These nanoemulsions are stable over months, even at high ${\varphi }$, and we did not observe any signs of droplet aggregation or coalescence at concentrations of NaCl below 100 mM used in our studies.We note that even for the most concentrated nanoemulsions we can assume that our droplets retain their nearly spherical shape. The bare volume fraction of the droplets does not exceed ${\varphi }\sim 0.7$ and thus droplet shape deformations due to packing effects are small, even for the highest concentrations. Equally, thermally excited shape fluctuations are negligible for SDS stabilized silicone oil droplets of such small size^38^.

The electrostatic double-layer repulsive interaction potential between the droplets can be approximated by $u(h)\simeq {C}_{{\rm{e}}}{e}^{-h/{\lambda }_{{\rm{D}}}}$ where $h=r-2R$ denotes the separation between surfaces of nearest neighboring droplets, $r$ is the center-to-center distance between these droplets, and ${C}_{{\rm{e}}}=2\pi R{\varepsilon }_{{\rm{r}}}{\varepsilon }_{0}{\psi }_{0}^{2}\gg {k}_{B}T$ is the contact potential energy for a surface potential ψ0 [mV]^39,40^. The Debye screening length of the nanoemulsion droplets ${\lambda }_{{\rm{D}}}=\sqrt{{\varepsilon }_{{\rm{r}}}{\varepsilon }_{0}{k}_{B}T\mathrm{/2}{e}^{2}I}$ is set by the total ionic strength $I$, given by the molar concentration of SDS plus the added NaCl. Here ${\varepsilon }_{0}$ is the vacuum permittivity and ${\varepsilon }_{{\rm{r}}}$ = 80 is the static relative dielectric constant of water. Over the electrolyte concentrations we examine, the nominal Debye length decreases from ${\lambda }_{{\rm{D}}}\simeq 3.1$ nm for 0 mM NaCl and 10 mM SDS to ${\lambda }_{{\rm{D}}}\simeq 1$ nm for 90 mM NaCl and 10 mM SDS. At very high ${\varphi }$, droplets become significantly deformed and eventually their elastic responses are governed by the Laplace pressure scale of the droplets $2\gamma /R$, where $\gamma$ is the surface tension^12,18^. Both the contact potential ${C}_{{\rm{e}}}$ and the surface tension can be affected by the addition of salt, see also Figure S2 (b) ^35,41^. Although we do not observe any signs of aggregation, at the highest NaCl concentrations, it is also possible that attractive van-der-Waals interactions $\sim {k}_{B}T$ can somewhat counteract the double-layer repulsion. Irrespective of these quantitative uncertainties, which may slightly shift the volume fractions associated with the glass transition and jamming transition, varying the ionic strengths of nanoemulsions through the NaCl concentration while fixing the SDS concentration provides a convenient means of accurately tuning droplet-droplet repulsive interactions over a significant range while also maintaining the stability of the nanoemulsion.

### Light scattering

**Figure 1:**
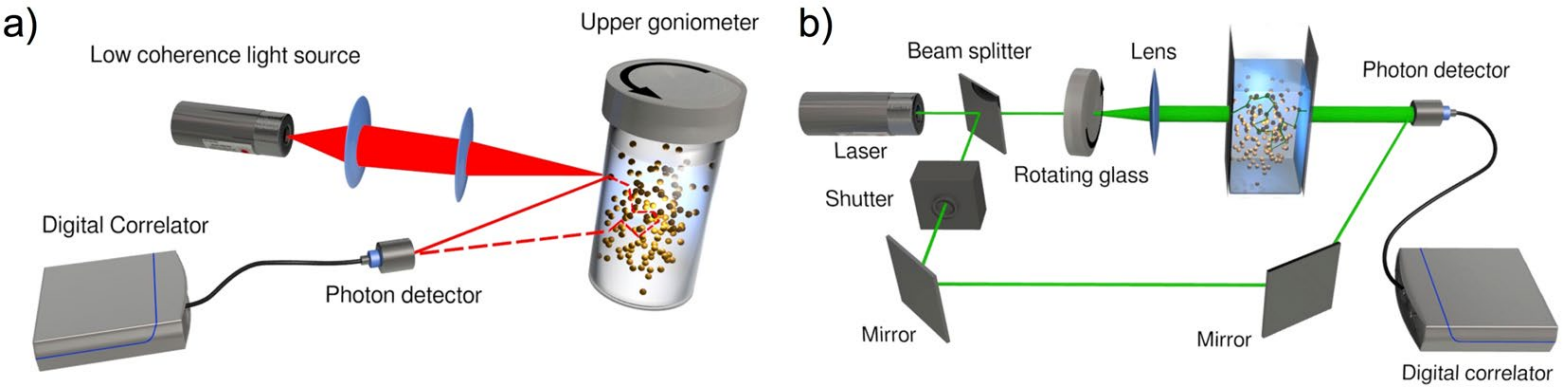
Experimental setups. (**a**) Schematic of the low-coherence dynamic light scattering (LC-DLS) apparatus. A low-coherence light source ${\lambda }_{\text{DLS}}=680$ nm (Superlum, Ireland), coherence length approximately $20\,\mu m$, is collimated and focused on the edge of the sample cell. The backscattered light is collected at an angle of $\theta \approx {170}^{\circ }$ with momentum transfer ${q}_{{\rm{DLS}}}\simeq 0.025$ nm^−1^ and analyzed by a digital correlator. An upper goniometer can be employed to rotate the sample and measure the ensemble averaged scattered intensity also for dynamically arrested samples. (**b**) Schematic of the diffusing wave spectroscopy (DWS) setup. A green (${\lambda }_{\text{DWS}}\,=\,532$ nm) laser light source is split into two beams. Two-cell Echo-DWS is implemented by scrambling the beam with a rotating ground glass diffuser^63^. The light is subsequently collimated to illuminate the sample with an approximately $8$ mm diameter beam. The multiply scattered light is detected by a photon detector in transmission. The second beam is acting at a local oscillator with adjustable intensity. A shutter allows for selection of homodyne or heterodyne configuration^42^.

The aim of our study is to characterize the internal dynamics and elasticity of disordered uniform nanoemulsions over a range of ${\varphi }$ extending below, through, and above both the glass transition and also the jamming transition. To this end, we employ finely tuned light scattering experiments to study the ensemble- and time-averaged dynamic scattering functions to extract the terminal relaxation times ${\tau }_{\alpha }$ and the plateau mean square displacements ${\delta }^{2}$ (MSDs) of droplets. To overcome challenges arising from the sample turbidity and the large dynamic range that we seek to cover, we use a combination of low-coherence dynamic light scattering (LC-DLS) near backscattering angles ($\theta \simeq {170}^{\circ }$) and heterodyne two-cell echo-diffusing wave spectroscopy (DWS)^42,43^, Figure 1. LC-DLS is sensitive to relatively large droplet displacements, appropriate for the near-glass regime below and around ${{\varphi }}_{{\rm{g}}}$, and at the same time LC-DLS efficiently suppresses the contributions of multiple scattering. Considering even larger ${\varphi }$, heterodyne DWS overlaps with LC-DLS and extends the measurements to very high concentrations where droplets become strongly jammed and begin to deform significantly. To ensure that we measure individual droplet MSDs and not other collective fluctuations we have carefully adjusted the experimental parameters such as droplet size, laser wavelength and the scattering configuration as explained in detail in the methods section.

### Monitoring the transition from the supercooled state to the glass

**Figure 2:**
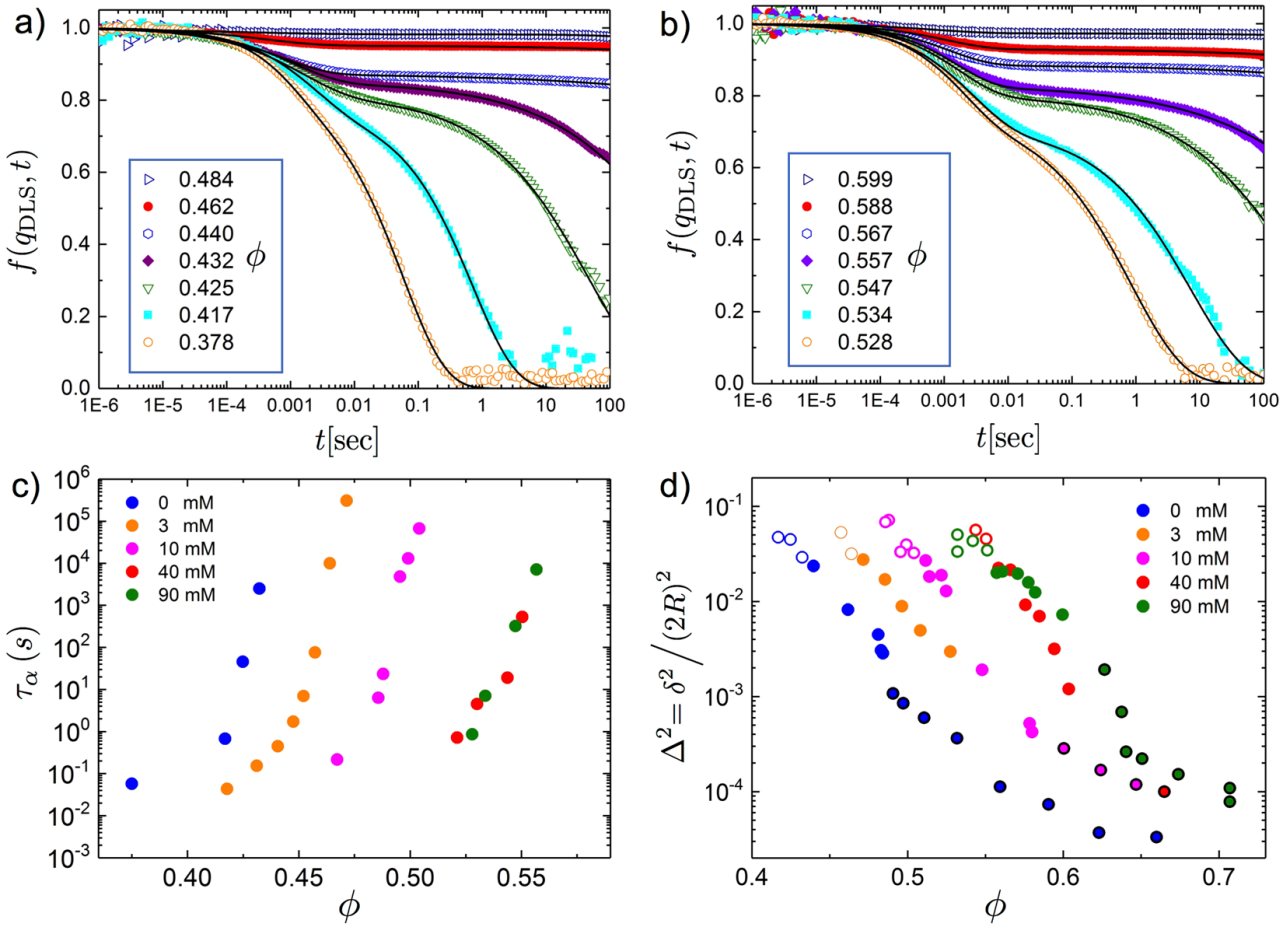
Light scattering and microscopic dynamic properties of dense nanoemulsions. Intermediate scattering functions (ISFs) of dense nanoemulsions composed of SDS stablized droplets with a radius $R=130$ nm for two different electrolyte concentrations: (**a**) 0 mM NaCl and (**b**) 90 mM NaCl. As the droplet volume fraction ${\varphi }$ increases, the ISFs develop pronounced double stretched exponential decays with the formation of an intermediate plateau having a value ${f}^{p}$, characteristic for a super-cooled liquid. Concurrently, the terminal relaxation time, called the $\alpha$-relaxation time, increases. Solids lines: double strechted-exponential fits (see text) (**c**) $\alpha$-relaxation time as a function of concentration for systems with different amounts of NaCl electrolyte added at fixed 10 mM SDS concentration. (**d**) Plateau values ${{\rm{\Delta }}}^{2}={\delta }^{2}\mathrm{/(2}R{)}^{2}$ of the mean square displacements (MSDs) derived from low-coherence dynamic light scattering (LC-DLS) and heterodyne diffusive wave spectroscopy (DWS). Open symbols denote MSD values for a transient plateau in the supercooled regime for ${\varphi } < {{\varphi }}_{{\rm{g}}}$ obtained from LC-DLS. Full symbols denotes data from LC-DLS for ${\varphi } > {{\varphi }}_{{\rm{g}}}$ and full symbols with a black circles are values derived from DWS. The error bars from the fits shown in a) and b) are smaller than the symbols shown in panel c) and d).

We present a set of normalized DLS intermediate scattering functions (ISFs) measured for nanoemulsions at different droplet densities with and without added NaCl, Fig. 2(a) and (b). We clearly observe the typical hallmarks of the glass transition: (1) a fast decay, known as the $\beta$-relaxation and the formation of an intermediate plateau ${f}^{p}$ and (2) the slow terminal relaxation of the ISF known as the $\alpha$-relaxation. Increasing the density, the $\alpha$-relaxation process becomes increasingly slow, extending to longer and longer times, until the onset of non-ergodicity occurs and the $\alpha$-relaxation no longer can be detected. For higher ionic strengths, both relaxations are shifted towards higher droplet ${\varphi }$ owing to the lower ${\lambda }_{{\rm{D}}}$. To quantify ${\tau }_{\beta },{\tau }_{\alpha }$ and ${f}^{p}$ we fit the normalized ISFs with a double stretched-exponential decay of the form: $f({q}_{{\rm{DLS}}},t)=\mathrm{(1}-{f}^{p}){e}^{-{(t/{\tau }_{\beta })}^{{b}_{1}}}+{f}^{p}{e}^{-{(t/{\tau }_{\alpha })}^{{b}_{2}}}$ ^28,44^. From the ISF plateau value ${f}^{p}={e}^{-{q}_{{\rm{DLS}}}^{2}{\delta }^{2}\mathrm{/6}}$ we can directly determine the plateau means square displacement ${\delta }^{2}$, Figs 2 and S4. The adjustable stretching parameters ${b}_{1},{b}_{2}$ are reported in the Supplemental Figure S3 for two electrolyte concentrations. Equally, from the DWS field correlation functions, shown in Figure S5, we obtain the MSD’s as explained in the methods section.

**Figure 3:**
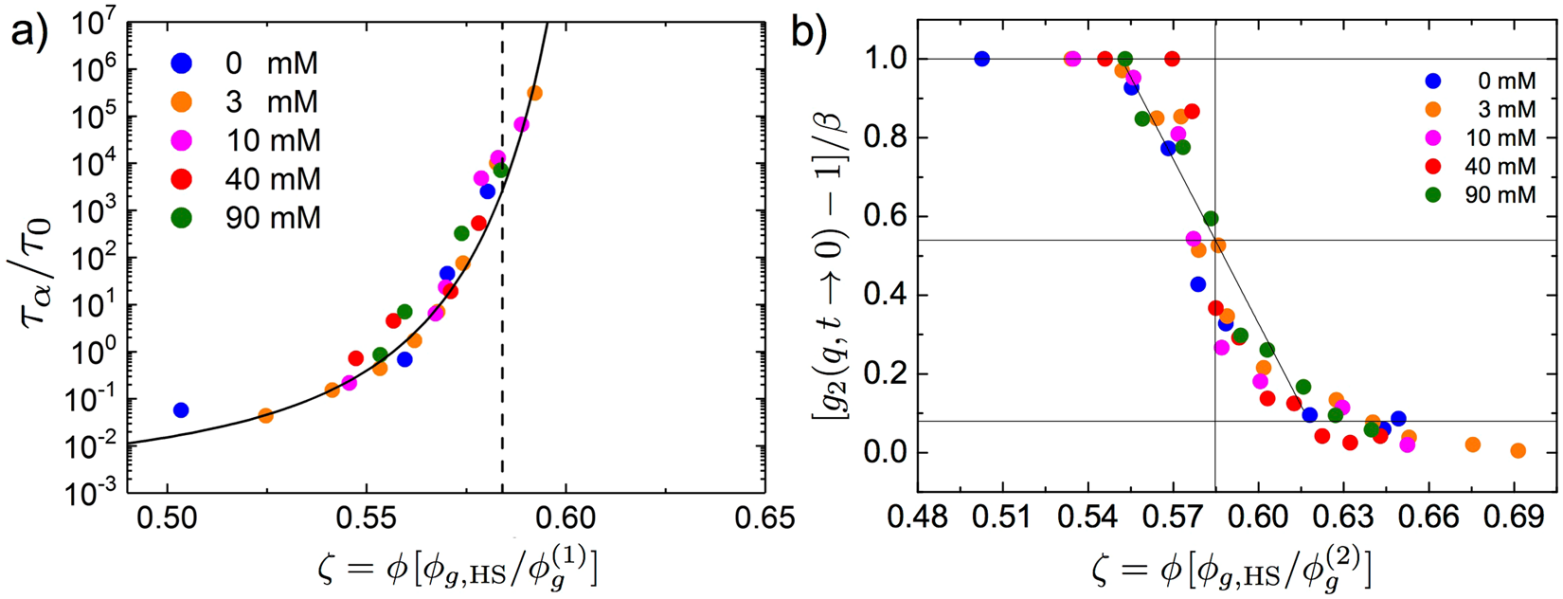
Determining the glass transition volume fraction. (**a**) Experimental values ${\tau }_{\alpha }$ rescaled onto a master curve by defining an effective packing fraction $\zeta =[{{\varphi }}_{g,\text{HS}}/{{\varphi }}_{{\rm{g}}}]{\varphi }$. Solid line: representing experimental data obtained for a hard sphere model system of PMMA colloids with similar size^28^, ${\tau }_{\alpha }/{\tau }_{0}={\tau }_{\infty }{e}^{A/({{\varphi }}_{{\rm{g}}}-{\varphi }{)}^{\delta }}$, with ${\tau }_{\infty }=1.54$ ms, $\delta =2$, $A=0.045$ and ${{\varphi }}_{\infty }=0.637$. ${\tau }_{0}=\mathrm{1/}{D}_{0}{q}^{2}=1.02$ ms is the decay time for a freely diffusing droplet having radius $R=130$ nm. ${D}_{0}$ is the Brownian diffusion constant^28^. (**b**) Decrease of intercept of the experimentally obtained ICF at the glass transition. ${{\varphi }}_{{\rm{g}}}^{\mathrm{(2)}}$ is determined as the concentration where $[{g}_{2}(q,t\to \mathrm{0)}-\mathrm{1]/}\beta$ has dropped by one half between $1$ and the minimum observed in the glass $[{g}_{2}(q,t\to \mathrm{0)}-\mathrm{1]/}\beta \sim 0.1$. $\beta$ denotes the coherence factor (see also Supplemental Figure S6).

Experimentally, it is challenging to objectively determine the glass transition volume fraction ${{\varphi }}_{{\rm{g}}}$. We thus apply three different procedures to estimate ${{\varphi }}_{{\rm{g}}}$ and compare the results. We first analyze the rapid increase of the relaxation time ${\tau }_{\alpha }$. In our realization of the DLS experiment we do not have access to the ultra-slow relaxation times that can be studied by other methods, such as multi-speckle DLS^28^. Moreover, the nanoemulsion droplet interactions are soft and thus our measurements can more easily be affected by experimental artifacts and secondary relaxations processes^31,45^. A straightforward way to overcome these limitations is to compare our experimental data for ${\tau }_{\alpha }$, covering a limited range of time scales, with previous, more extensive, measurements on hard spheres. As shown in Fig. 3(a) all our data for the $\alpha$-relaxation time collapse on a master curve for hard spheres, taken from^28^, by rescaling the ${\varphi }$-axis with a factor ${{\varphi }}_{g,\text{HS}}/{{\varphi }}_{{\rm{g}}}^{\mathrm{(1)}}$ (${{\varphi }}_{g,\text{HS}}:=0.58$)^1,4^. The only adjustable parameter is ${{\varphi }}_{{\rm{g}}}^{\mathrm{(1)}}$, reported in Table 1 and Figure S7. Next we study the decrease of intercept of the experimental intensity-intensity correlation function ${g}_{2}(q,t\to \mathrm{0)}$ to determine the onset of the glass. For an ergodic liquid system, the time averages probed in DLS also correspond to ensemble averages taken over all possible system configurations. In this case the well known Siegert relation can be applied and thus $f(q,t)=\sqrt{({g}_{2}(q,t)-\mathrm{1)/}\beta }$, where $\beta$ is the coherence factor adjusted to normalize $f(q,t\to \mathrm{0)}=1$. Its value is determined experimentally and it depends on the properties of the probing light beam and on the geometrical alignment of the DLS-instrument. However, for samples that have reached the glass state the measured ICF drops and $({g}_{2}(q,t)-\mathrm{1)/}\beta$ can be used as a sensitive probe to determine the glass transition volume fraction. The value ${{\varphi }}_{{\rm{g}}}^{\mathrm{(2)}}$ can be estimated from a linear fit to $({g}_{2}(q,t)-\mathrm{1)/}\beta$. As shown in Fig. 3b), when plotted against the effective volume fractions, the concentration dependence is the same for all ionic strengths and is in full agreement with data previously reported by Bartsch and coworkers^4,31^. As a third estimate we define as ${{\varphi }}_{{\rm{g}}}^{\mathrm{(3)}}$ the highest droplet concentration that exhibits a decay of $f(t)$ by more than 10% below the plateau value ${f}^{p}$ within a correlation time window up to 100 seconds. The results from these three procedure are reported in Table 1. Within experimental error ${\rm{\Delta }}{\varphi }\sim 0.005$ all three procedures yield essentially identical results.

**Table 1:** Comparison of the experimental glass transition volume fraction obtained from three different methods. (i) ${{\varphi }}_{{\rm{g}}}^{\mathrm{(1)}}$: obtained by adjusting the effective packing fraction $\zeta =[{{\varphi }}_{g,\text{HS}}/{{\varphi }}_{{\rm{g}}}^{\mathrm{(1)}}]{\varphi }$ to map the measured ${\tau }_{\alpha }$ on literature results for model hard spheres as shown in Fig. 3(a). (ii) ${{\varphi }}_{{\rm{g}}}^{\mathrm{(2)}}$: determined from the onset of nonergodicity in the DLS experiment, Fig. 3(b), (iii) ${{\varphi }}_{{\rm{g}}}^{\mathrm{(3)}}$: derived from the first concentration value where $f({q}_{{\rm{DLS}}},t)\ge 0.9$ over all $t < 100$ s. See also Supplemental Figure S7.

NaCl	0 mM	3 mM	10 mM	40 mM	90 mM
${{\varphi }}_{g}^{\mathrm{(1)}}$	0.433	0.467	0.510	0.557	0.557
${{\varphi }}_{g}^{\mathrm{(2)}}$	0.436	0.456	0.510	0.557	0.557
${{\varphi }}_{g}^{\mathrm{(3)}}$	0.432	0.472	0.504	0.558	0.567

Our results demonstrate that while approaching the glass phase from the supercooled liquid side the dynamics of a nanoemulsion systems follows the glass transition scenario for hard spheres, provided the volume fraction is renormalized, taking into account the screened double-layer repulsion. Similar observations have been reported previously for the the glass transition of charged silica colloids^46^. Equivalently, in numerical studies, it was shown that the crystallization transition of charged spheres can be mapped to the hard-sphere case in the limit of short range interactions ${\lambda }_{{\rm{D}}}\ll R$. An effective packing fraction $\zeta =[{{\varphi }}_{g,\text{HS}}/{{\varphi }}_{{\rm{g}}}]{\varphi }$ can be defined using by a shift factor ${{\varphi }}_{g,\text{HS}}/{{\varphi }}_{{\rm{g}}} > 1$ and the latter depends on the electrolyte concentrations $I$. Despite the close similarities with hard spheres when approaching ${{\varphi }}_{{\rm{g}}}$, it is important to point out that, in contrast to the hard-sphere case, the plateau value ${f}^{p}$ increases slightly when approaching the glass transition^2,4,28^. This increase is clearly visible in Fig. 2(a) and (b). When approaching ${{\varphi }}_{{\rm{g}}}$ the size of the transient cages shrinks but at the same time droplet-droplet interactions also slightly stiffen, owing to the concentration dependence of the repulsive double-layer potential. Finally, from the ${f}^{p}$-values and from DWS we extract the plateau values of the MSD, shown in Fig. 2(d), penetrating deep into the glass and jamming regimes. The range of MSDs covered by our light scattering experiments, more than three order of magnitude, is remarkable.

### The glass-jamming transition scenario for charged nanoemulsion droplets

**Figure 4:**
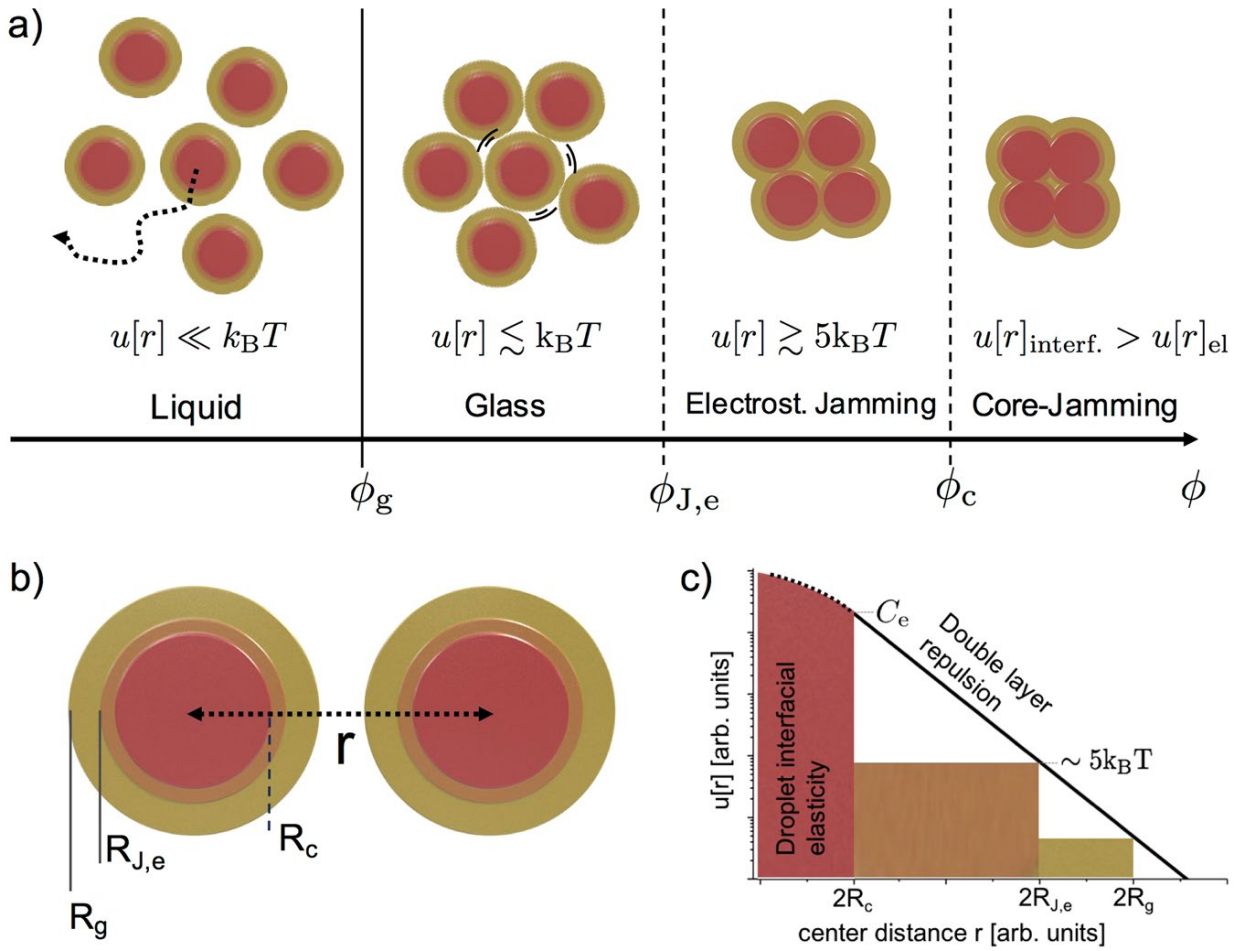
Different stages of the glass-jamming transition for charged nanodroplets. (**a**) Schematic representation of transition from the liquid to glass and to jammed states. (a) The entropic glass-transition (solid line), in the weak interaction limit $u[r]\mathop{ < }\limits_{ \tilde {}}{{\rm{k}}}_{{\rm{B}}}T$, is well defined at ${{\varphi }}_{{\rm{g}}}$. The jamming transitions at ${{\varphi }}_{{\rm{J}},{\rm{e}}}$ and ${{\varphi }}_{{\rm{c}}}$, denoted by dashed lines, are smeared. (**b**) Characteristic distances $2{R}_{{\rm{x}}}$ between neighboring droplets when crossing the glass and the jamming transitions in nanoemulsions. (**c**) Repulsive interaction energy on a logarithmic scale plotted as a function of the mean distance between droplets $r$.

For volume fractions ${\varphi } > {{\varphi }}_{{\rm{g}}}$ our nanoemulsions are dynamically arrested and behave as soft solids. When further increasing the packing fraction the plateau values of the MSD’s drop sharply, signaling that the droplet motion becomes more and more spatially constrained. For soft spheres, in the presence of finite range repulsive interactions, there is a competition between energetic and entropic contributions when minimizing the free energy. To illustrate the coupling between the increase in interaction energy and the vanishing free volume we first discuss some important typical length scales, Fig. 4. We also note that for hard spheres the available free volume at the glass transition can be used to define a radius ${R}_{g} > R$ slightly larger than the hard sphere radius $R$ with ${R}_{g}/R\simeq \sqrt[3]{{{\varphi }}_{{\rm{J}}}/{{\varphi }}_{{\rm{g}}}}=\sqrt[3]{1.1}$. The typical distance a particle can move in the cage formed by it’s neighbours is then ${R}_{g}-R$.

For soft and finite ranged droplet interactions, the pair-distances and packing fractions where direct interactions become dominant are not as well defined as for hard spheres. Nonetheless, the exponential increase of the double-layer repulsion still sets a rather well defined threshold volume fraction ${{\varphi }}_{{\rm{J}},{\rm{e}}}$ where $u[r=2{R}_{{\rm{J}},{\rm{e}}}] > c\,\cdot \,{k}_{B}T$. Here $c$ is a factor we expect to be larger than one but less than 10. A change of volume fraction by $0.01$ close to ${{\varphi }}_{{\rm{J}},{\rm{e}}}$ corresponds to a change in interaction energy by several ${k}_{B}T$ and thus ${{\varphi }}_{{\rm{J}},{\rm{e}}}$ only depends very weakly on the exact choice of $c$. Additionally, the capacity of the droplet interfaces to deform elastically defines a third characteristic length scale $\sim {R}_{c}$ and packing fraction ${{\varphi }}_{c}$, corresponding to the ‘core’ interfacial deformation controlled by the surface tension of the droplets. Typically the droplets are relatively stiff and thus interfacial deformation only sets in once the double-layer has been compressed strongly and the elastic contribution becomes comparable to the contact potential energy ${C}_{e}$.

In summary, we can divide the behavior of charged nanoemulsions into four characteristic regimes: i) the liquid and supercooled near-glass regime ${\varphi } < {{\varphi }}_{{\rm{g}}}$, ii) the glass regime ${{\varphi }}_{{\rm{g}}} < {\varphi } < {{\varphi }}_{{\rm{J}},{\rm{e}}}$, iii) the electrostatic jamming regime ${{\varphi }}_{J,e} < {\varphi } < {{\varphi }}_{{\rm{c}}}$ corresponding to significant overlap of the Debye layer, yet very little droplet interfacial deformation, and iv) the interfacial jamming regime ${\varphi } > {{\varphi }}_{{\rm{c}}}$. The latter we call ‘core-jamming’ at ${{\varphi }}_{c}$. Depending on the size of the droplets, their elasticity and the range and strength of the double-layer repulsion as well as polydispersity, all of these transitions could be more or less smeared.

For the classical case of micron scale droplets the transition regime ${{\varphi }}_{{\rm{J}},{\rm{e}}} < {\varphi } < {{\varphi }}_{{\rm{c}}}$ is reduced to a small interval of a few percentile. For droplets of this size it has been shown that the zero frequency elastic modulus increases as ${G}_{{\rm{p}}}\simeq \mathrm{1.6(}\gamma /R){\varphi }({\varphi }-{{\varphi }}_{{\rm{c}}})$ for ${\varphi } > {{\varphi }}_{{\rm{c}}}$ where $\gamma$ is the droplet surface tension^12,24^. However, for the charged nanomulsions studied in here, the radius is about an order of magnitude smaller. Since entropic contributions to the local elastic modulus scale as ${k}_{B}T/{R}^{3}$ their weight is significantly increased. Moreover, due to the smaller radius the transitions intervals become sizable, while the interactions remain short-ranged ${\lambda }_{{\rm{D}}}\ll R$. Therefore, by studying nanoscale emulsions, we are able to probe and resolve the dynamic properties over all the different stages of the liquid-glass-jamming transition.

### The glass-jamming transition: model comparison

For a quantitative interpretation, we compare our light scattering data, Fig. 2(d), to a quasi-equilibrium thermodynamic EEI model for the plateau shear modulus ${G}_{{\rm{p}}}({\varphi })$ of colloidal emulsions^18,19,35,47^. This EEI model reproduces the main features of the smooth crossover between glassy entropic scale to the jammed interfacial scale of colloidal emulsions through near-equilibrium free energy minimization^19,47^, mediated by screened electrostatic repulsions^18,48^. Such a smooth crossover is also effectively incorporated into a unified scenario for soft disordered materials, as proposed by Ikeda *et al*. through simulations^7,49^. The EEI model has successfully been used to describe ${G}_{{\rm{p}}}({\varphi })$ of charge stabilized microscale and nanoscale emulsions, measured using macroscopic shear rheometry, from the glassy regime across the jamming transition, including the effects of screened electrostatic repulsions^19,35^. However, a quantitative comparison of the EEI model to light scattering experiments covering the entire ${\varphi }$-range of interest, encompassing the liquid-glass and the glass-jamming transition, has not been reported yet.

**Figure 5:**
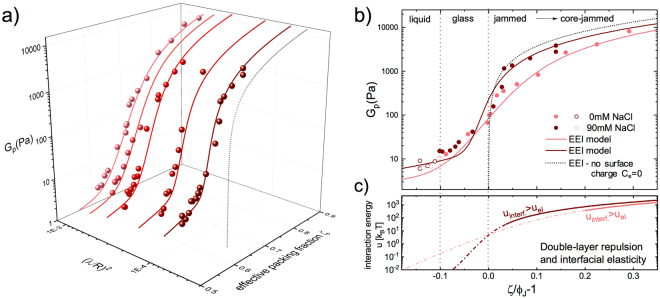
Shear modulus ${G}_{p}$ of charged nanoemulsions across the glass and the jamming regimes. (**a**) Symbols: full data sets derived from all the MSD-data shown in Fig. 2(d), below and above ${{\varphi }}_{{\rm{g}}}$, and plotted as a function of the effective packing fraction $\zeta$ for different ionic strengths $I$ with ${({\lambda }_{{\rm{D}}}/R)}^{2}=({\varepsilon }_{{\rm{r}}}{\varepsilon }_{0}{k}_{B}T\mathrm{/2}{R}^{2}{e}^{2})\times {I}^{-1}$. Lines show the predictions by the EEI model with a constant surface potential ${\psi }_{0}=230$ mV and otherwise the same parameters reported in^35^. (**b**) Open symbols: data for ${\varphi } < {{\varphi }}_{{\rm{g}}}$ where ${{\rm{\Delta }}}^{2}$ and the plateau modulus ${G}_{p}$ are transient. Full symbols: data for ${\varphi } > {{\varphi }}_{{\rm{g}}}$. $\zeta =\mathrm{(0.58/}{{\varphi }}_{{\rm{g}}}^{\mathrm{(1)}}){\varphi }$ for the experimental data and $\zeta =({{\varphi }}_{{\rm{J}}}/{{\varphi }}_{{\rm{J}},{\rm{e}}}){\varphi }$ for the EEI model calculations, with ${{\varphi }}_{{\rm{J}}}=0.646$. We extract ${{\varphi }}_{{\rm{J}},{\rm{e}}}$ from the EEI calculations as the concentration where $u[{k}_{B}T]=5$. The dotted lines shows the prediction in the athermal limit (${k}_{B}T/{R}^{3}\to 0$) with ${G}_{p}\simeq \mathrm{1.6(}\gamma /R)\zeta (\zeta -{\phi }_{J})$ and $\gamma =6.6$ mN/m^12,35^. The vertical dash-dotted lines indicate the position of the jamming $\zeta /{\phi }_{{\rm{J}}}-1=0$ and the glass transition $\zeta /{\phi }_{{\rm{J}}}-1\simeq -0.1$ for ideal hard spheres. (**c**) Lines: Droplet-droplet interaction energy $u[{k}_{B}T]$. Solid lines denote the regime where interfacial elasticity exceeds the electric double-layer repulsion.

Clearly, light scattering offers several advantages over mechanical rheometry: high sensitivity and very large dynamic ranges in both time and MSD values. To compare our light scattering data to the EEI model for ${G}_{{\rm{p}}}({\varphi })$, we convert the particle’s plateau MSD ${\delta }^{2}$ to ${G}_{{\rm{p}}}$ using a model based on single-particle microrheology^50,51^ of a harmonically bound Brownian particle (HBBP)^52,53^. In the continuum HBBP microrheological framework, an equation connecting ${\delta }^{2}$ to ${G}_{{\rm{p}}}$ is easily derived from the generalized Stokes-Einstein relation^50^, evaluated in the plateau region: $\pi R{\delta }^{2}{G}_{{\rm{p}}}={k}_{B}T$. A system composed of nearly identical discrete particles which also serve as the probes, such as the droplets in a colloidal emulsion, does not exactly satisfy the continuum assumption, so the validity of this microrheological relationship is not expected to hold exactly, although it has been demonstrated to provide reasonable approximate predictions in many practical applications for which the continuum assumption isn’t strictly satisfied^54^. Thus, the existence of some numerical correction factor between the model’s predictions and the MSDs obtained from light scattering would not be surprising. In the following, we use ${G}_{{\rm{p}}}=0.3{\rm{Pa}}/{{\rm{\Delta }}}^{2}$ which corresponds to twice the value predicted for a HBBP ${G}_{{\rm{p}}}={k}_{B}T/\pi R{\delta }^{2}=0.15{\rm{Pa}}/{{\rm{\Delta }}}^{2}$, with ${{\rm{\Delta }}}^{2}={\delta }^{2}\mathrm{/(2}R{)}^{2}$ and ${k}_{B}T/{R}^{3}=1.85$ Pa for $T={22}^{\circ }$ C and $R=130$ nm. The excellent agreement between the model and the experimental data is shown in Fig. 5(a) and (b). All results are are plotted as a function of the effective packing fraction, $\zeta =\mathrm{(0.58/}{{\varphi }}_{{\rm{g}}}^{\mathrm{(1)}}){\varphi }$ for the experimental data and $\zeta =\mathrm{(0.646/}{{\varphi }}_{{\rm{J}},{\rm{e}}}){\varphi }$ for the EEI model calculations. At ${\varphi }$ below and slightly above the glass transition, $\zeta /{{\varphi }}_{{\rm{J}}}-1\sim -0.1$, the data for the nanoemulsion systems with 0 mM and the system with 90 mM NaCl superimpose, Fig. 5(b), signaling that this regime is insensitive to the characteristics of the interaction potential and the properties are governed by hard-sphere-like entropic contributions. Above the glass transition packing fraction the increase in ${G}_{{\rm{p}}}$ become more steep, which is most evident for the sample with 90 mM NaCl, Fig. 5(b). The inflection point of this accelerated increase approximately matches the prediction for jamming at $\zeta =0.646$, which we identify as the vestige of the ideal jamming transition that occurs when the effective radii ${R}_{{\rm{J}},{\rm{e}}}$ of the charged nanoemulsions begin to overlap and the mean square displacements are getting substantially smaller. For these higher concentrations $\zeta /{\phi }_{{\rm{J}}}-1 > 0$ we also observe a notable difference between data obtained in the presence and absence of added NaCl.

The EEI model is based on a quasi-equilibrium free energy minimization and couples all three contributions to the free energy, namely entropic, electrostatic and interfacial. Thus, from the comparison of the experimental data with the EEI model we can extract the relative contributions and differentiate the different regimes. To distinguish the glass and the jamming regime we plot the mean droplet-droplet interaction energy $u$, which is the sum of the double layer repulsion and the interfacial deformation energy, for each packing fraction ${\varphi }$ (Fig. 5(c)). Around the glass transition, $\zeta /{{\varphi }}_{{\rm{J}}}-1=-0.1$ the interaction energy is very small $u\ll {k}_{B}T$. Direct interactions increase from there on and eventually dominate when $u$ clearly exceeds the thermal energy. We have taken $u=5{k}_{B}T$ to define the threshold value ${{\varphi }}_{{\rm{J}},{\rm{e}}}$ which corresponds to $\zeta /{{\varphi }}_{{\rm{J}}}-1=0$. Further increasing the packing fraction the interfacial energy increases more strongly and eventually exceeds the double layer repulsions. For the 90 mM Nacl sample this happens much earlier than for the 0 mM sample. Both the transition at ${{\varphi }}_{{\rm{J}},{\rm{e}}}$ and ${{\varphi }}_{{\rm{c}}}$ are continuous and smeared together with the glass transition. As shown here, the different regimes cannot be easily identified by studying ${{\rm{\Delta }}}^{2}({\varphi })$ or ${G}_{p}({\varphi })$ alone and can only be disentangled once we have identified the different contributions to the free energy.

## Summary and Conclusions

The collapse of our ${G}_{{\rm{p}}}$-data in the vicinity of ${{\varphi }}_{{\rm{g}}}$ and the observed differences around the jamming transition support the generic scenario for the glass and the jamming transition in soft sphere suspensions^7^. In such a unified description of the glass and jamming transition both are entangled and the transitions are smeared together. Only for potentials that are sufficiently stiff can the entropic- and the enthalpic-dominated regimes be identified clearly. In our system, we have chosen relatively small droplets that can be studied by light scattering and that would also display a readily measurable plateau shear modulus near and above the glass transition, ${k}_{B}T/{R}^{3} > 1Pa$. Moreover, as a consequence of the small droplet size, direct interactions are dominated by electrostatic repulsive double-layer forces over a relatively large range of concentrations, whereas interactions due the interfacial elasticity of the emulsion droplets only play a dominant role deep in the (core-)jammed regime. Based on our finely tuned light scattering measurements we have precisely mapped out the dependence of the droplet thermal motion on the electrolyte concentration. By adding NaCl, we cause the double-layer interaction potential $u(r)\simeq {C}_{e}{e}^{-(r-2R)/{\lambda }_{{\rm{D}}}}$ to become stiffer. The effective spring constant or bond strength $k$ of pairwise interactions, given by the second derivative of the pair potential $u(r)$, is proportional to the ionic strength $k\propto 1/{\lambda }_{D}^{2}\propto I$ ^18^. ${k}^{-1}$ is also inversely proportional to the effective temperature ${T}_{e}={k}_{B}T\mathrm{/(4}{R}^{2})\times {k}^{-1}$ commonly used as a measure of particle softness in simulations^7,49^ and separating the different regimes in a unified glass-jamming phase digram for soft spheres. Here, we have varied the total ionic strength $I$ by one order of magnitude, from 10 mM to 100 mM for a typical effective temperature ${T}_{e} \sim ({\lambda }_{D}/R{)}^{2}(1/2c)$ at *ϕ*_(J,e)_ (where *u* = *c* *k*_*B*_ *T* and *c*~5) and therefore, in our experiments the effective temperature is decreasing from approximately ${10}^{-4}$ to $-{10}^{-5}$. Our data thus covers the interesting transition regime between soft and hard spheres and lends strong support to previous simulation results^7^. Our comparison of light scattering data and the EEI-model also shows that the glass and the jamming regimes are distinct and that the transition can be gradual and smooth, or more step-like, depending on the softness of the interaction potential between droplet surfaces.

## Methods

### Nanoemulsions

We produce a polydisperse nanoemulsion using a high pressure microfluidic device. This nanoemulsion is subsequently size-fractionated and osmotically concentrated by a series of ultracentrifugation steps^34,48,55^. The nanoemulsion is composed of poly-dimethylsiloxane (PDMS, Gelest, viscosity 10 cSt, mass density 0.935 g/cm^3^), SDS (MP Biomedicals, ultrapure), and deionized water (Millipore MilliQ Academic, resistivity 17 M${\rm{\Omega }}$ cm). A microscale premix emulsion at ${\varphi }\mathrm{=0.3}$ and [SDS] = 20 mM is first subjected to 3 passes through a high pressure homogenizer (Microfluidics Inc., Microfluidizer 110-P, 75 $\mu$m Y-type interaction chamber, water-ice bath cooled) at a peak liquid pressure of 10,000 psi $\,\simeq \,$ 69 MPa. The resulting emulsion of droplets having much smaller sizes is subsequently diluted to ${\varphi }=0.15$ using an aqueous solution at [SDS] = 10 mM. Three ultracentrifugal fractionation steps (Beckman L8-55 Ultracentrifuge, SW-28 swinging bucket rotor, polycarbonate tubes) are then used, redispersing the same fractions of the solid plugs in from different tubes 10 mM SDS solution to ${\varphi }\simeq 0.15$ after each step except the last one: 15,000 rpm for 9 hours (retaining the top 1/2 of the solid plug), 13,000 rpm for 8 hours (retaining the top 1/3 of the solid plug), and 12,000 rpm for 8 hours (retaining middle third of the solid plug). Throughout this fractionation process, the SDS concentration is maintained at 10 mM, in excess of SDS’s critical micelle concentration of $\simeq$8 mM, to ensure stabilization against droplet coalescence. A deeply jammed master nanoemulsion sample is prepared with an elastic modulus ${G}_{{\rm{p}}}$ much larger than 1 kPa, Figure S1. This master sample is then used to prepare a concentration series by means of dilution, fixing the final SDS concentration to always be 10 mM, with sensitivity of $\sim 0.005$ in ${\varphi }$. Separate nanoemulsion samples, each having a different NaCl concentration, were then created by diluting the master sample with the desired ionic strength (while maintaining the SDS concentration at 10 mM) and afterwards centrifuging again to reach an elastic jammed state at high ${\varphi }$. Before the beginning of each light scattering experiment, each sample was prepared by following procedure. First, the entire sample was stirred for few minutes until the sample was thoroughly mixed and homogenized. Then, gentle centrifugation was applied to fill the entire cell uniformly and to remove small bubbles that were occasionally found to be trapped inside the sample.

### Characterization of droplet size and polydispersity

We determine the size and polydispersity of the droplets by conventional dynamic light scattering at λ = 532 nm under highly diluted conditions. An exact determination of the small but finite polydispersity is crucial for this study, but it is also notoriously difficult based on DLS data alone. In order to measure the polydispersity precisely we apply the method of Pusey and Segré by measuring the apparent droplet hydrodynamic radius for different scattering angles or $$q$$-vectors as described in^56,57^. For each $$q$$ vector the hydrodynamic radius is calculated from the first cumulant of the correlation function^57^. The results are shown in the Supplemental Figure S2(a). First the apparent radius is decreasing, until it reaches a minimum value from where the apparent radius increases quickly reaching a new maximum. The $$q$$-dependence of the hydrodynamic radius is a consequence of the partial masking of scattering from the subpopulation of particles, where the minimum of the scattering form factor exactly matches the $$q$$-value selected. For spherical scatterers and for scattering in the Rayleigh-Gans-Debye limit ($$2\pi {n}_{s}({n}_{p}/{n}_{s}-\mathrm{1)}a/\lambda \sim 0.1\ll 1$$) the width of this curve provides a sensitive measure of the polydispersity $$\sigma $$ with a precision of about $$\mathrm{1 \% }$$. In the Supplemental Figure S2 we plot the ratio between the apparent radius obtained in the light scattering device, divided by the real radius obtain as extrapolation of the apparent radius to zero momentum transfer $$q$$-. This analysis results in a mean size or $$ < R > =130$$ nm and a polydipersity of the system of $$\mathrm{12(}\pm \mathrm{1) \% }$$, nearly identical values as for the hard-sphere-like sample studied in^28^. We note that the slight overshooting at large $$q$$-values can be explained by the increased instrument error at large scattering angles $$\Theta  > {130}^{\circ }$$ including errors due to contributions of light scattered at small angles but subsequently reflected inside the cuvette^58^.

### Light scattering experiments

The aim of our study is to characterize the the internal dynamics and elasticity of disordered uniform colloidal emulsions from the liquid, through the near-glass and full glassy regime corresponding to dynamic arrest, and well into the highly compressed jammed states at even larger ${\varphi }$. In order to realize this goal, we use a particular variant of dynamic light scattering and also diffusing wave spectroscopy. Although rheological data on uniform colloidal emulsions already cover these regimes, light scattering offers important advantages over mechanical rheometry. The amount of sample needed for each light scattering measurement is relatively little, of the order of few hundreds of microliters at all ${\varphi }$; whereas, for mechanical rheometry, very large sample volumes of up to several milliliters are typically required at lower ${\varphi }$ in order to boost torque signals at small strains into a measurable range. In our experiments this is important because it enables us to obtain a high resolution in ${\varphi }$ of light scattering data below, through, and above both glass and jamming transitions using only a limited quantity of the same fractionated master nanoemulsion sample. Moreover, the samples prepared for light scattering experiments measurement can be kept in a sealed cuvette which strictly eliminates evaporation of the solvent and thus allows hour long measurments and a fine control of the concentration of samples under examination with a precision ${\rm{\Delta }}{\varphi }\le 0.005$ and better. The challenges in using light scattering for this purposes are twofold. First, our samples are strongly scattering in the visible range of optical wavelengths, so a careful design of the light scattering experiments is required. Second, we aim to cover an unusually large range of droplet displacements for the arrested states. To address these challenges, we use a combination of low-coherence dynamic light scattering (LC-DLS) near backscattering angles (${\rm{\Theta }}\simeq 170$) and heterodyne diffusing wave spectroscopy (DWS), Figure 1. Low-coherence dynamic light scattering is sensitive to relatively large droplet displacements, covering the liquid regime and the glass transition; at the same time, LC-DLS efficiently reduces the contributions of multiple scattering, thereby facilitating quantitative interpretation in terms of single droplet dynamics. Heterodyne DWS overlaps with LC-DLS in the desired ${\varphi }$-range that we explore and extends the measurements to very high concentrations.

At volume fractions above $0.3$, disordered colloidal nanoemulsions display typical short range order positional correlations^34,59^. In reciprocal space the ordering manifests itself through the emergence of a pronounced peak in the structure factor $S(q)$ at a peak wave number ${q}_{m}\simeq 2\pi /d$ where $d$ is the typical center-to-center separation between particles in the first correlation shell. For $q\ll {q}_{m}$ light scattering probes the collective decay of density fluctuations; whereas, for $q\ge {q}_{m}$ structural relaxations and self-motion are probed^60^. In our experiments we aim to probe the latter, $q\ge {q}_{m}$, using both LC-DLS and DWS. The typical particle-particle separation is always larger than $d\sim 2R=260$ nm and thus ${q}_{m}\le 2\pi \mathrm{/(2}R)$. We operate the DLS experiment nearly under backscattering conditions $sin(\theta \mathrm{/2)}\simeq 1$ and thus ${q}_{{\rm{D}}{\rm{L}}{\rm{S}}}\simeq 2k=4\pi /{\lambda }_{\text{DLS}}$ where ${\lambda }_{\text{DLS}}=680\,nm/1.33=511$ nm is the wavelength in water and k = 2π/λ the wavenumber in water. Therefore ${q}_{{\rm{DLS}}}/{q}_{m}\ge 1$ is fulfilled. For DWS, all scattering vectors $q=0\to 2k$ contribute, but the measured dynamic scattering function is determined by a ${q}^{3}$-weighted average over $f(q,t)$ which gives a strong bias to higher $q$-values approaching ${q}_{\max ,\text{DWS}}=2k=4\pi /{\lambda }_{{\rm{DWS}}}$. We have chosen a wavelength for the DWS laser to be about 25% lower compared to that of the LC-DLS setup in order to ensure a dominant contribution of wave vectors $q\ge {q}_{m}$. Thus, collective contributions do not significantly affect the measured DWS signal which is dominated by the single-droplet MSD, corresponding to the self-motion of individual droplets driven by thermal fluctuations.

We custom-made a LC-DLS apparatus, Figure 1, that is able to measure mean square displacements in the vicinity of the glass transition for highly multiply scattering systems. To this end, we illuminate the sample cuvette with a super luminescent diode with low coherence length ($lc\approx 20\,\mu m$) and we detect the back-scattered light by positioning the detector at an angle $\theta \approx 170$. In this near-backscattering configuration, the singly back-scattered light contributes coherently to the intensity correlation function, whereas the light that has undergone multiple scattering and has traveled for a distance larger than the coherence length, cannot interfere at the detector and thus does not contribute to the intensity correlation function. In the concentration regime studied the scattering mean free path ${l}_{s}\simeq 200\,\mu m$ and the transport mean free path ${l}^{\ast }\simeq 500\,\mu m$ are much larger than the coherence length of the light source. As a result, the intensity correlation function is determined by single scattering contributions only. Since the amount of the incoherent multiple scattered light is higher than the coherent single scattering contribution, the intercept of the intensity correlation function is strongly reduced, typically of the order of few ${10}^{-2}$. In order to maximize the intercept, the beam is focused with a lens with short focal length at the cuvette wall as shown in Figure 1(a). With an additional sample goniometer we slowly rotate the sample cuvette. The intermediate scattering function (ISF) can then be properly derived from the measured intensity correlation function (ICF) ${g}_{2}(q,t$) using common procedures as described in^61^. Prior to the measurement, the ICF of the system at rest is recorded several times (in time intervals of 10 minutes) to ensure that the system shows no signs of aging but has reached a steady state, see also Supplemental Figure S8.

Diffusing wave spectroscopy (DWS) allows us to probe very small MSDs that are not accessibly by LC-DLS. From the measured DWS correlation function, we extract the field correlation function and the average droplet MSD as described in^42^ ${g}_{1,\text{DWS}}=x/\sinh \,[x]$, $x=(L/{l}^{\ast })\sqrt{{k}^{2}{\rm{\Delta }}{\overrightarrow{r}}^{2}(t)}$. $L$ is the cuvette path length (typically $L=5$ mm, width $10$ mm), $k=2\pi /{\lambda }_{{\rm{DWS}}}$ the wave number in water and ${l}^{\ast }$ the optical transport mean free path in the multiple scattering sample^62^. The latter is determined experimentally by comparison of the diffuse optical transmission intensity with a sample of known ${l}^{\ast }$. In order to have sufficient overlap between DWS and LC-DLS we have implemented DWS with partial heterodyning, as has been previously described^42,43^. In the strong heterodyning limit, the DWS experiment accesses directly the field intermediate scattering function ${g}_{1,\text{DWS}}(t)$; whereas, in the homodyne limit, it accesses the correlation function ${g}_{2}(t)=1+\beta {g}_{1,\text{DWS}}{(t)}^{2}$. This heterodyne DWS approach allows us to expand the range of accessible MSDs by about one order of magnitude. To obtain a heterodyne measurement, we split off part of the main beam using a beam splitter, and we redirect it to the detector with two mirrors. A shutter and an intensity attenuator enable switching between heterodyne and homodyne configurations by either blocking the local oscillator or adjusting the power of the local oscillator, Figure 1.

### Modeling

Predictions for ${G}_{{\rm{p}}}({\varphi })$ of ionic colloidal emulsions are calculated using an entropic, electrostatic, interfacial (EEI) model based on quasi-equilibrium free energy minimization that includes and couples all three contributions through a common droplet deformation parameter^35^. A shear strain $\gamma$ is introduced into this three-term free energy quadratically, thereby enabling the calculation of ${G}_{{\rm{p}}}({\varphi })$ consistent with free energy minimization, which is accomplished using the Mathematica (Wolfram Research, Inc., Champaign, IL, USA) FindRoot function. At each ${\varphi }$, a quadratic least-squares fit is performed on the $\gamma$-dependent minimized free energies, yielding the curvature of the free energy with respect to the strain at $\gamma =0$, from which ${G}_{{\rm{p}}}$ is determined.

The contact potential ${C}_{e}=u(h=\mathrm{0)}$, which enters the double layer repulsive contributions in the electrostatic term of the EEI model as a prefactor, is difficult to determine precisely experimentally since it can be influenced by the added NaCl electrolyte concentration^27,35^. This is supported by electrophoretic mobility measurements on the nanoemulsions included in the supplementary information (Figure S2 (b). Moreover we believe it is possible that residual van-der-Waals attractions potentially contribute at sub-nanoscale distances, partially compensating for the double layer repulsion. The exact magnitudes of these potential influences are difficult to quantify, however. Obtaining an accurate measurement of the contact potential arising from the equilibrium adsorbed surfactant concentration on the surfaces of the droplets is very difficult, as it cannot be easily inferred from traditional zeta potential measurements. We have thus chosen to compare the model’s predictions with experiments using reduced volume fractions. We define the reduced volume fraction by rescaling the bare volume fraction ${\varphi }$ with the factor $\mathrm{0.646/}{{\varphi }}_{{\rm{J}},{\rm{e}}}$ where ${{\varphi }}_{{\rm{J}},{\rm{e}}}$ denotes the droplet volume fraction where interaction energy exceeds $u=5{k}_{B}T$. Thus $\zeta =0.646$ corresponds to ${\varphi }={{\varphi }}_{J,e}$. By plotting the predictions as a function of $\zeta$ we largely eliminate the dependence of the EEI on the choice of the double-layer contact potential ${C}_{e}$, see also Figure S9. For the EEI calculations shown in Fig. 5 we use ${\psi }_{0}\sim 230$ mV and thus ${C}_{e}=7.5\times {10}^{3}{k}_{B}T$. The ratio $\mathrm{0.646/}{{\varphi }}_{{\rm{J}},{\rm{e}}}$ is the only additional global shifting parameter we introduce. We note that in the limit ${\lambda }_{D}/R\to 0$ we recover $\zeta ={\varphi }$.

### Data availability

Data needed to evaluate the conclusions in the paper are present in the paper and/or the Supplementary Materials. Additional data related to this work may be requested from the authors.

## Electronic supplementary material

Supplementary Material
