# Supplementary material for: The liquid-glass-jamming transition in disordered ionic nanoemulsions
Source: Sci Rep. 2017 Nov 8;7:13879. doi: 10.1038/s41598-017-13584-w (PMC5678350; doi:10.1038/s41598-017-13584-w)
Supplement: Supplementary file 1 — Supplementary Material [file 41598_2017_13584_MOESM1_ESM.pdf]

Supplementary Information: The liquid-glass-jamming transition in disordered ionic nanoemulsions

Marco Braibanti,[1] Ha Seong Kim,[2] Nesrin Şenbil,[1] Matthew J. Pagenkopp,[2] Thomas G. Mason,[3] and Frank Scheffold[1]

[1] *Department of Physics, University of Fribourg, CH-1700 Fribourg, Switzerland*
[2] *Department of Chemistry and Biochemistry, University of California, Los Angeles, California 90095, USA*
[3] *Department of Chemistry and Biochemistry, and Department of Physics and Astronomy, University of California, Los Angeles, California 90095, USA*

(Dated: October 8, 2017)

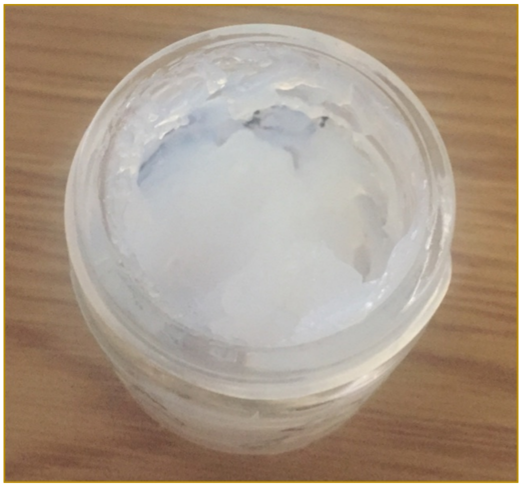

FIG. S1. **Translucent visual appearance of the concentrated nanoemulsions.** Image of the concentrated stock of nanoemulsion in a vial. The emulsion appears optically translucent and has a paste like consistency.

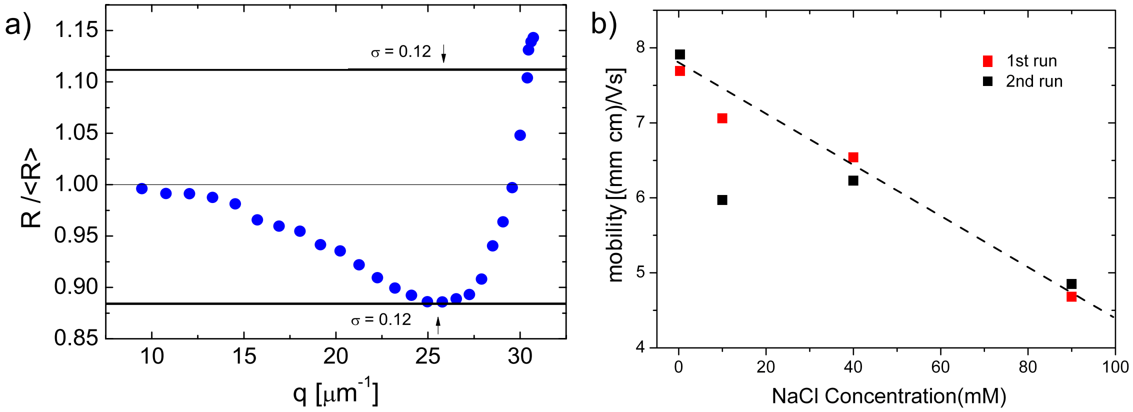

FIG. S2. **Characterization of highly diluted nanoemulsion droplets in water.** a) Size characterization of the nanoemulsion droplets: apparent hydrodynamic radius R from dynamic light scattering measurements (ALV, Germany, $\lambda = 532$nm) as a function of the momentum transfer q. R decreases with increasing q until it reaches a minimum value of 0.88 $< R >$ and then it sharply increases, reaching values of about 1.12 $< R >$. For each q vector the hydrodynamic radius is calculated from the first cumulant of the correlation function. The mean hydrodynamic radius can be obtained from the low-q limit to $< R >= 130 \pm 2$ nm. From the width of the curve we extract a polydispersity of $\sigma = 12\%$. b) Electrophoretic mobility (DelsaMax, Coulter & Beckman, USA) in units of [(mm cm)/V s] of the droplets as a function of the NaCl electrolyte concentration. The instrument is specified for a maximum conductivity of 50mS/cm and the sample with highest ionic strength (90mM NaCL) has a conductivity of approximately 10 mS/cm.

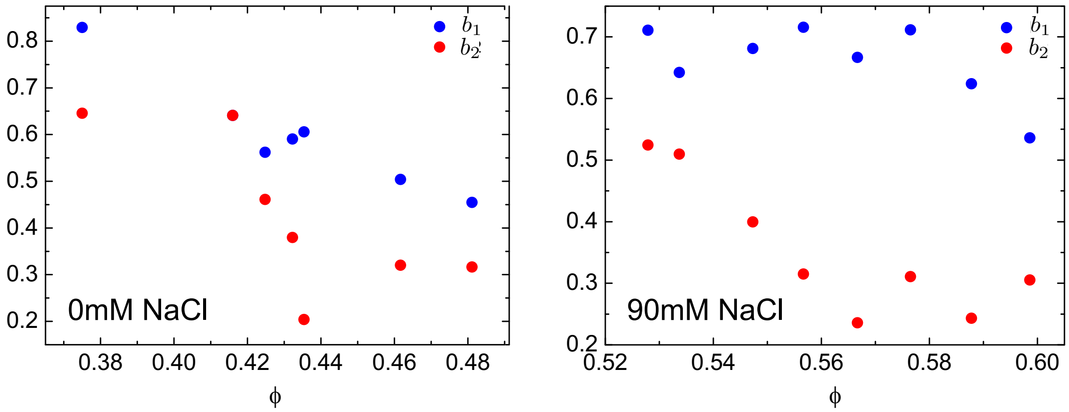

FIG. S3. **Stretching exponents of the ISF when approaching the glass.** Stretching exponents obtained from the fit of $f(q_{\rm DLS},t) = (1-f^p)e^{-(t/\tau_\beta)^{b_1}} + f^p e^{-(t/\tau_\alpha)^{b_2}}$ to the intermediate scattering functions for two different electrolyte concentrations.

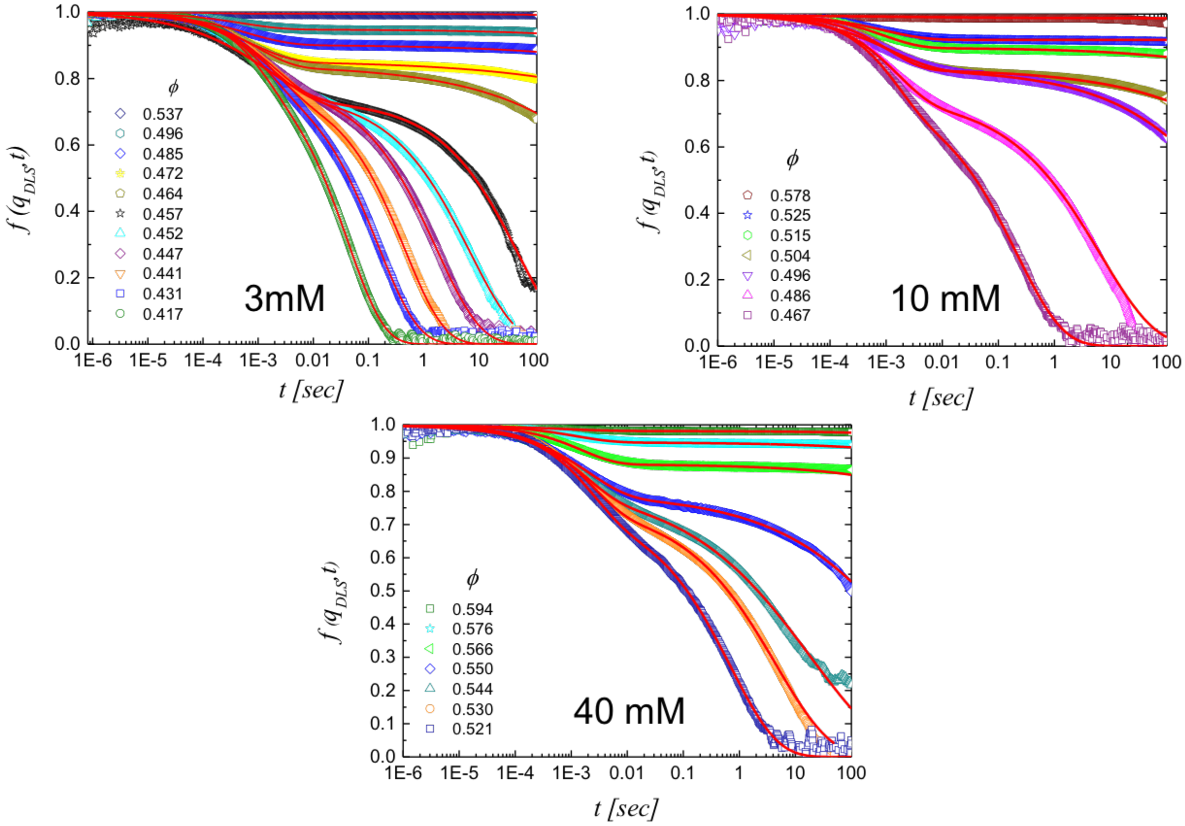

FIG. S4. **Intermediate scattering functions (ISFs)**. $f(q,t)$ at different concentrations for the three NaCl electrolyte concentrations: 3 mM ,10mM and 40mM NaCl, not shown in the main text.

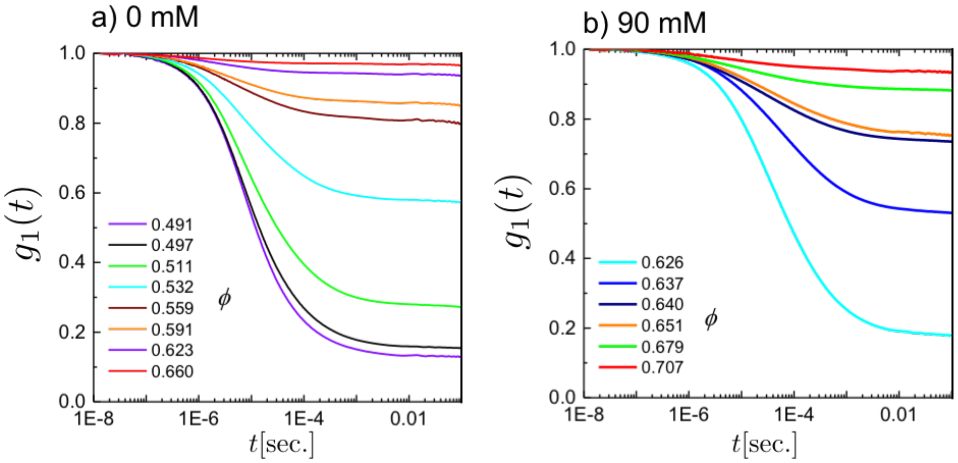

FIG. S5. **Field correlation functions from two-cell DWS with partial heterodyning.** $g_1(t)$ at different concentrations for two electrolyte concentrations 0mM and 90mM.

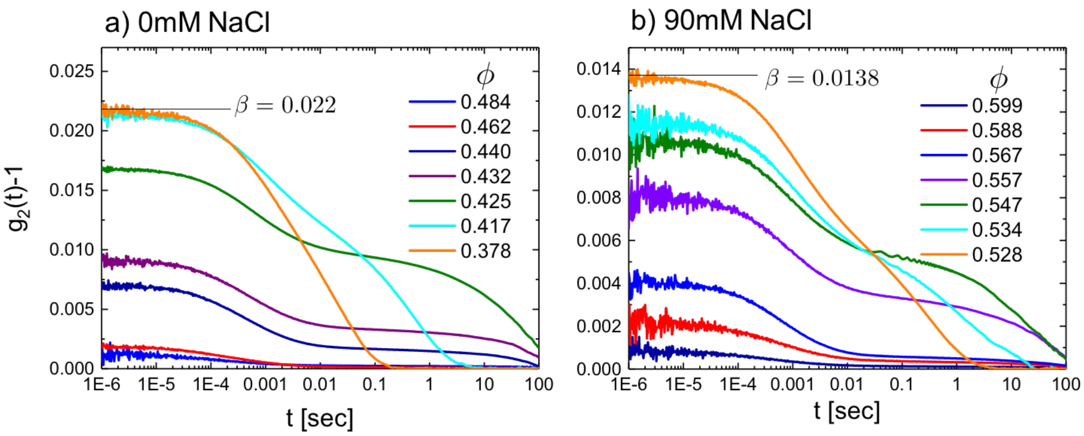

FIG. S6. **Decay of the intercept of the ICF when entering the non-ergodic glass regime.** Intensity correlation functions $g_2(t) - 1$ from LC-DLS across the glass transition for a) 0mM NaCl and b) 90mM NaCl.

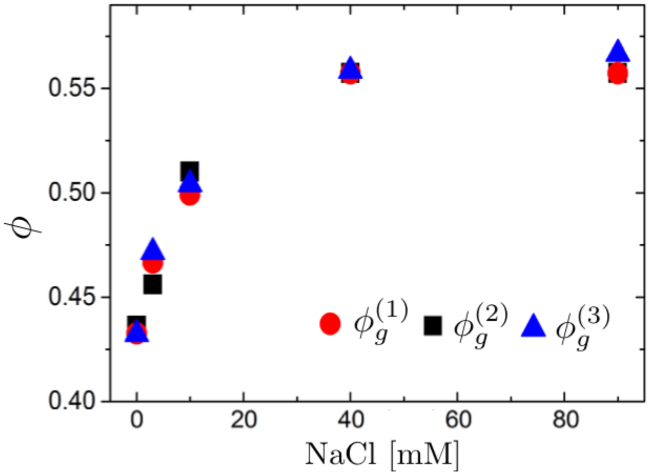

FIG. S7. **Glass transition volume fractions for different ionic strengths.** Plot of the values reported in table 1.

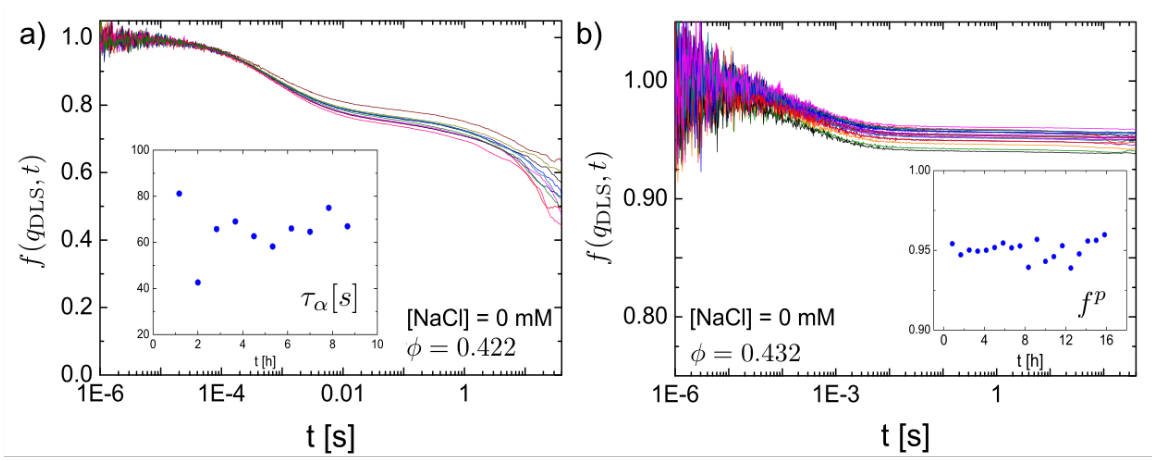

FIG. S8. **Control experiments to verify that results are not affected by aging.** Intermediate scattering functions recorded recorded in succession over 10 minutes time intervals and for two different ϕ values at 0 mM NaCl. The measurements were started after an initial waiting time of typically about 30 min to 1 hour has passed. Both decay time τ_α and intermediate plateau height f^p do not evolve in time, indicating that the sample does not age appreciably over time scales of an hour and has reached a steady state before the light scattering measurements have begun.

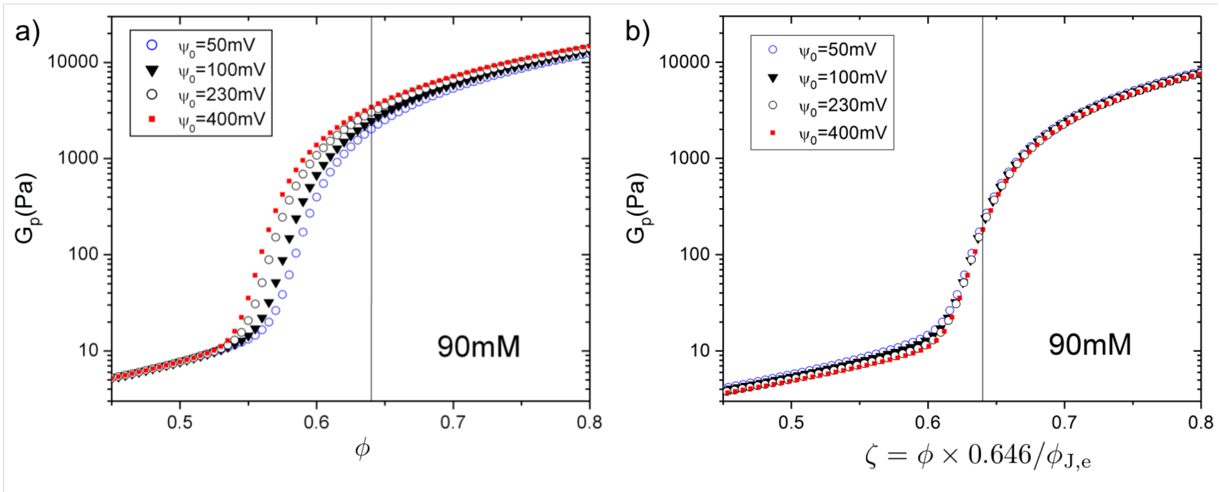

FIG. S9. **Illustration how rescaling on an effective packing fraction ζ makes G_p insensitive to the exact choice of the surface potential $C_e \propto \psi_0^2$.** By rescaling on an effective packing fraction the G_p-predictions of the EEI model for different values of the surface potential ψ_0 at constant ionic strength shown in a) collapse onto a master curve b). The effective packing fraction is defined as $\zeta := [\phi_{\rm J}/\phi_{\rm J,e}]\,\phi$ by setting $\phi_{\rm J,e} = \phi$ exactly when $u = 5k_BT$. Solid line: $\zeta = 0.646$.
